# Prostaglandin E_2_ inhibits matrix mineralization by human bone marrow stromal cell-derived osteoblasts via Epac-dependent cAMP signaling

**DOI:** 10.1038/s41598-017-02650-y

**Published:** 2017-05-22

**Authors:** Ali Mirsaidi, André N. Tiaden, Peter J. Richards

**Affiliations:** 1 Bone and Stem Cell Research Group, CABMM, University of Zurich, 8057 Zurich, Switzerland; 2 Zurich Center for Integrative Human Physiology (ZIHP), University of Zurich, 8057 Zurich, Switzerland

## Abstract

The osteoinductive properties of prostaglandin E_2_ (PGE_2_) and its signaling pathways have led to suggestions that it may serve as a potential therapeutic strategy for bone loss. However, the prominence of PGE_2_ as an inducer of bone formation is attributed primarily to findings from studies using rodent models. In the current study, we investigated the effects of PGE_2_ on human bone marrow stromal cell (hBMSC) lineage commitment and determined its mode of action. We demonstrated that PGE_2_ treatment of hBMSCs significantly altered the expression profile of several genes associated with osteoblast differentiation (*RUNX2* and *ALP*) and maturation (*BGLAP* and *MGP*). This was attributed to the activation of specific PGE_2_ receptors, and was associated with increases in cAMP production and sustained AKT phosphorylation. Pharmacological inhibition of exchange protein directly activated by cAMP (Epac), but not protein kinase A (PKA), recovered the mineralization functions of hBMSC-derived osteoblasts treated with PGE_2_ and restored AKT phosphorylation, along with the expression levels of *RUNX2*, *ALP*, *BGLAP* and *MGP*. Our findings therefore provide insights into how PGE_2_ influences hBMSC-mediated matrix mineralization, and should be taken into account when evaluating the role of PGE_2_ in human bone metabolism.

## Introduction

Prostaglandins are lipid metabolites derived from arachidonic acid through the actions of cyclooxygenase (COX)-1 and COX-2, and display a diverse range of functions in numerous biological systems including cardiovascular, renal, gastrointestinal, respiratory, reproductive, neurologic and musculoskeletal^1, 2^. Prostaglandin E_2_ (PGE_2_) is by far the most well studied of the prostanoids, mediating its effects via four G protein-coupled receptor subtypes, designated as EP1-4^3^. EP1 acts to induce calcium influx and enhance intracellular free calcium^4^. EP2 and EP4 are predominantly involved in mediating increases in cAMP levels, while the primary function of EP3 is to inhibit cAMP production^5^.

It is has long been established that PGE_2_ plays an important role in regulating bone metabolism^6–8^, although there is still some debate as to whether its primary mode of action is to promote bone formation or bone resorption^9, 10^. Insights into the potential signaling pathways regulating PGE_2_ mediated bone turnover have been gleaned from studies utilizing mice deficient in specific PGE_2_ receptors, the results from which have identified PGE_2_ receptor subtypes EP2 and EP4 as being central players in the maintenance of a normal bone phenotype^11, 12^.

The capacity for PGE_2_ to enhance bone formation has largely been attributed to its stimulatory effects on bone marrow stromal cell (BMSC) osteogenesis^13–15^. However, findings from *in vitro* studies utilizing either rat BMSCs or human adipose-derived stromal cells suggest that PGE_2_ may also have a negative influence on osteogenesis^16, 17^. More recently, it has been shown that PGE_2_ has the capacity to facilitate human BMSC (hBMSC) adipogenesis at the expense of osteogenesis, and that these effects were associated with the enhanced expression of PGE_2_ receptors EP2 and EP4 in response to dexamethasone treatment^18^. Such effects may be of clinical relevance when considering the detrimental effects of long-term dexamethasone therapy on human bone quality^19^. Indeed, both clinical and experimental investigations have provided evidence to suggest that osteogenesis is impaired in dexamethasone-induced osteoporosis, while adipogenesis is enhanced^20, 21^.

In the present study, we set out to further evaluate the influence of PGE_2_ on hBMSC lineage commitment, and to provide a more in-depth assessment of its mode of action by focusing primarily on the signaling pathways through which PGE_2_ mediates its effects. We demonstrated that PGE_2_ significantly compromised the ability of hBMSC-derived bone forming cells to mineralize matrix *in vitro* in a dose dependent manner, being primarily regulated by the EP2/4-cAMP-Epac signaling pathway. The negative impact of PGE_2_ on hBMSC-mediated bone formation was further highlighted by its ability to stimulate hBMSC adipogenesis under conditions conducive to either osteogenic or adipogenic differentiation.

## Results

### Influence of PGE_2_ on hBMSC osteogenesis and adipogenesis

**Figure 1:**
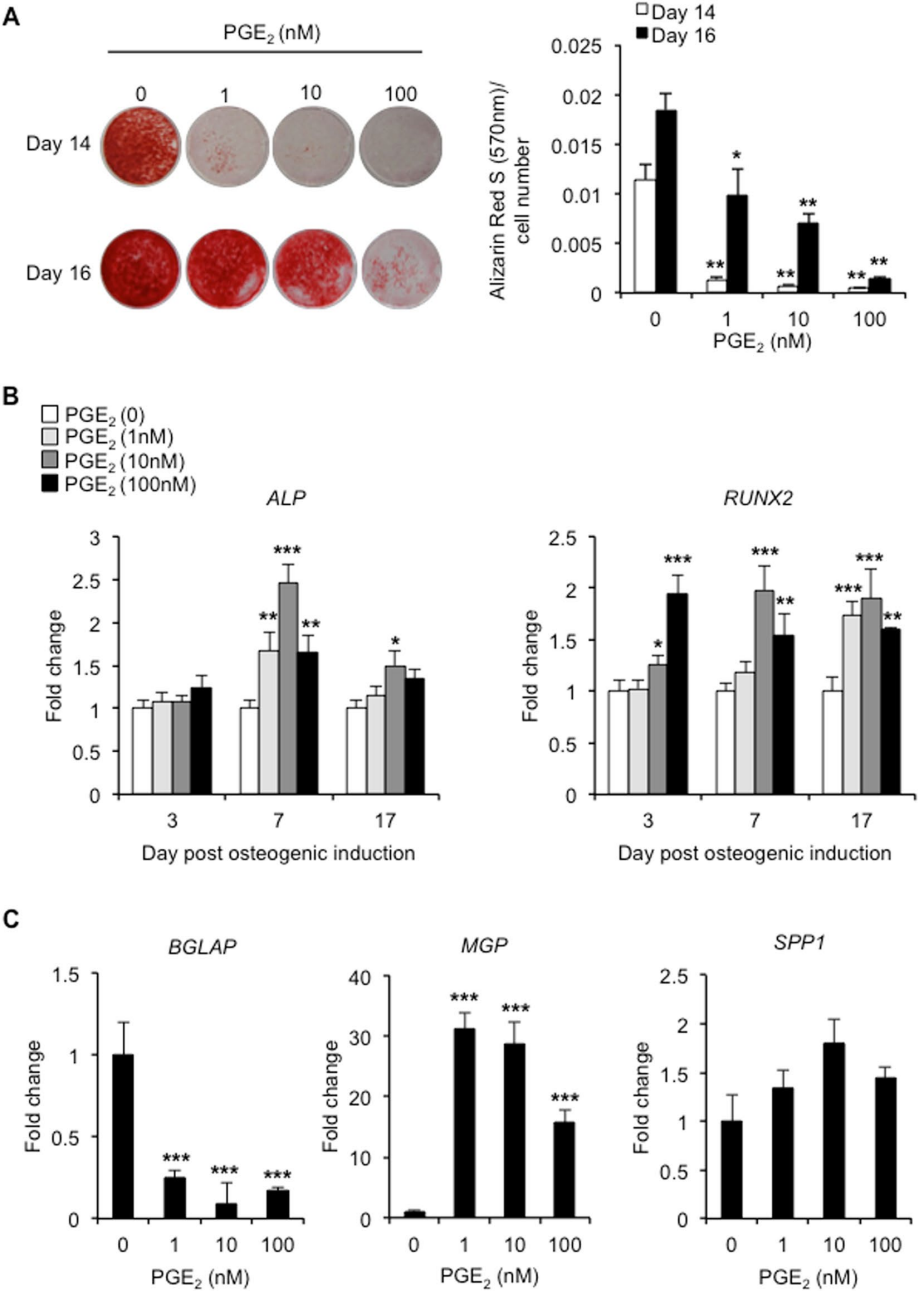
PGE_2_ inhibits hBMSC-mediated matrix mineralization. (**A**) Alizarin Red S staining was used to assess the influence of continuous PGE_2_ treatment on matrix mineralization in hBMSC cultures at 14 and 16 days post-osteogenic induction. *p < 0.01, **p < 0.001 as compared to untreated hBMSCs using ANOVA. (**B**,**C**) RT-qPCR was used to determine expression levels of osteogenic differentiation markers *RUNX2* and *ALP* at day 3, 7 and 17 post-osteogenic induction (**B**), and markers of osteoblast maturation and/or matrix mineralization *BGLAP*, *MGP* and *SPP1* at day 17 post-osteogenic induction (**C**). Data were normalized to *GUSB* and expressed as fold change as compared to non-induced controls at day 0 (value 1) using the comparative *C*_T_ method. *p < 0.05, **p < 0.01, ***p < 0.001 as compared to untreated hBMSCs using ANOVA. The data represent triplicate determinations and were replicated at least two times. All values are presented as mean ± S.D.

Alizarin Red S staining of mineralized matrix was used to assess the effects of prostaglandin treatment on hBMSC-derived osteoblast development. Long-term exposure of hBMSCs to PGE_2_ impaired their ability to generate functional osteoblasts in a dose-dependent manner as evidenced by significant reductions in Alizarin Red S staining after 14 and 16 days of osteogenic differentiation (Fig. 1A). These effects were also observed in BMSCs harvested from two other human donors (Supplementary Fig. 1). We also examined the effects of the closely related prostaglandin PGD_2_ on hBMSC-derived osteoblast mineralization, but found its inhibitory actions to be greatly diminished as compared to PGE_2_ (Supplementary Fig. 2). In order to assess whether the inhibitory effects of PGE_2_ were also evident at the molecular level, we measured the expression levels of various osteogenic markers using RT-qPCR. Despite the marked inhibitory actions of PGE_2_ on BMSC-mediated matrix mineralization, we failed to observe any reductions in the expression levels of osteogenic differentiation markers runt-related transcription factor 2 (*RUNX2*) and alkaline phosphatase (*ALP*) at early (day 3 and 7) and late (day 17) stages of osteogenesis (Fig. 1B). To the contrary, the expression levels of both genes were significantly increased in response to PGE_2_ treatment at early and late time points. Attempts were also made to determine the expression levels of Osterix (*SP7*), but values remained below detection limits. We next investigated whether PGE_2_ treatment had any influence on the expression of gene markers directly involved in regulating osteoblast maturation and/or matrix mineralization. Indeed, expression levels of the osteoblast-specific marker osteocalcin (*BGLAP*) were significantly decreased in cultures at day 17 following treatment with PGE_2_ (Fig. 1C). By contrast, expression levels of the potent inhibitor of calcification matrix gla protein (*MGP*), were significantly enhanced in PGE_2_-treated hBMSCs. Moderate increases in osteopontin (*SPP1*) expression levels were also observed, although statistical significance was not attained. Attempts were also made to measure the expression levels of osteocyte markers *SOST* and *DMP1*. However, in both cases, expression levels remained below detection limits. Based on these initial findings, we selected PGE_2_ at a concentration of 10 nM for further studies.

**Figure 2:**
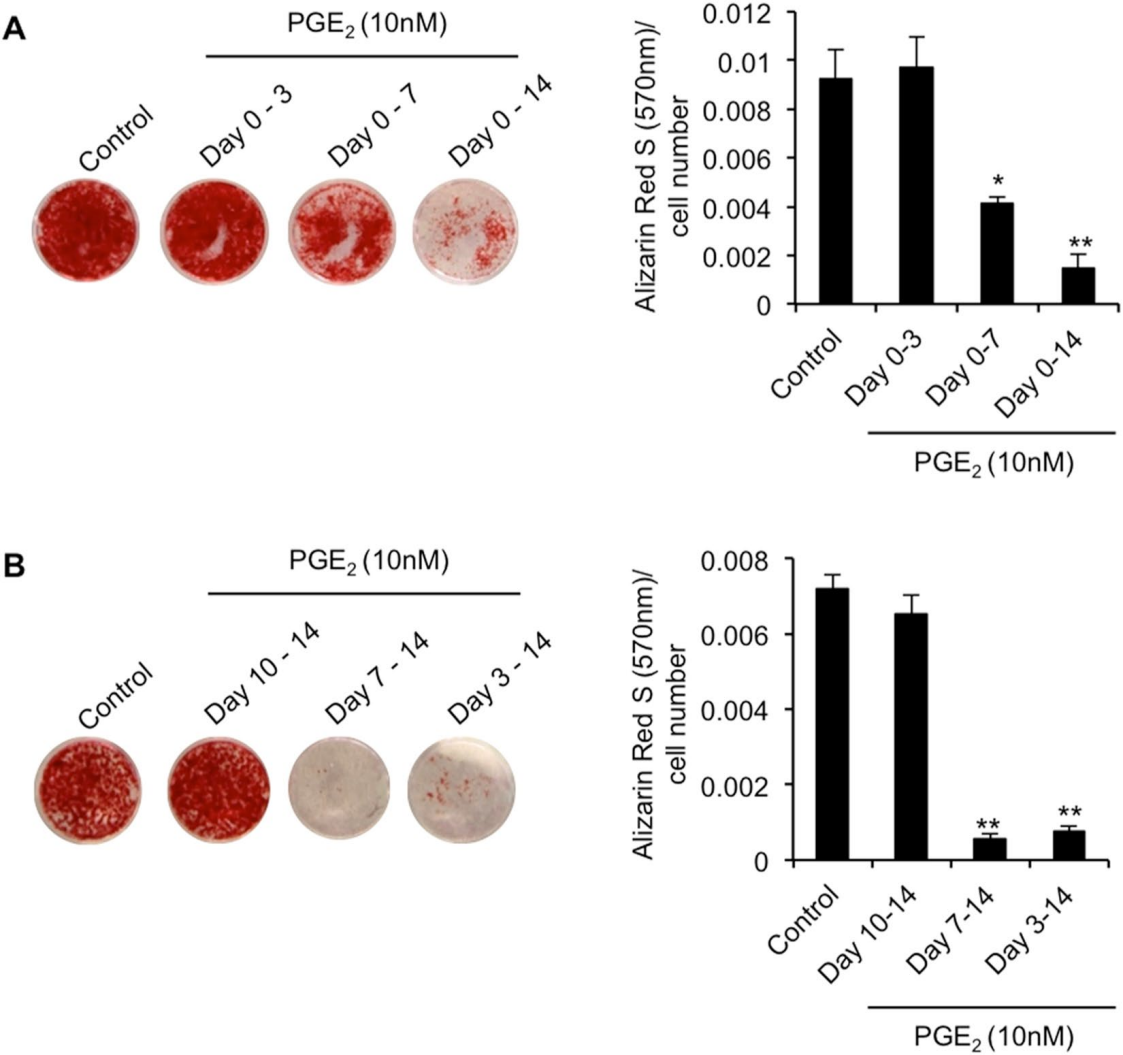
Effect of PGE_2_ on hBMSC-mediated matrix mineralization is dependent on differentiation stage of hBMSC osteogenesis and duration of exposure. hBMSCs were treated with PGE_2_ (10 nM) for varying durations beginning at the initiation of osteogenic induction (Day 0) (**A**), or at selected time points thereafter (**B**), and matrix mineralization quantified at day 16 by Alizarin Red S staining. *p < 0.01, **p < 0.001 as compared to hBMSCs induced to undergo osteogenesis for 14 days in the absence of PGE_2_ (control) using ANOVA. The data represent triplicate determinations and were replicated at least two times. All values are presented as mean ± S.D.

Due to the apparent differential effects of PGE_2_ on the expression of early (*ALP*, *RUNX2*) and late (*BGLAP*) osteogenic markers in differentiating hBMSCs, we surmised that the inhibitory actions of PGE_2_ on matrix mineralization may be related to its ability to influence hBMSC-derived osteoblast maturation, rather than hBMSC osteogenic differentiation *per se*. To investigate this, we next examined whether the time point at which PGE_2_ was added to hBMSCs, and its duration of exposure, had any influence on its ability to inhibit matrix mineralization by hBMSC-derived osteoblasts. hBMSCs were induced to undergo osteogenic differentiation for 14 days, and treated with PGE_2_ for varying durations staring either at the time of induction (Fig. 2A), or at various time points thereafter (Fig. 2B). Our findings demonstrated that an exposure time of at least 7 days was required for PGE_2_ to elicit an inhibitory effect on mineralized matrix formation, and that PGE_2_ was equally effective whether added to cells at the time of osteogenic induction, or 7 days later. These results therefore support the concept that PGE_2_ most likely inhibits matrix mineralization through its ability to impair the function of hBMSCs already committed to osteoblasts, and that its stimulatory influence on early markers of osteogenic differentiation is not sufficient to overcome these effects, and may actually prevent hBMSC-derived osteoblasts from reaching terminal maturation.

**Figure 3:**
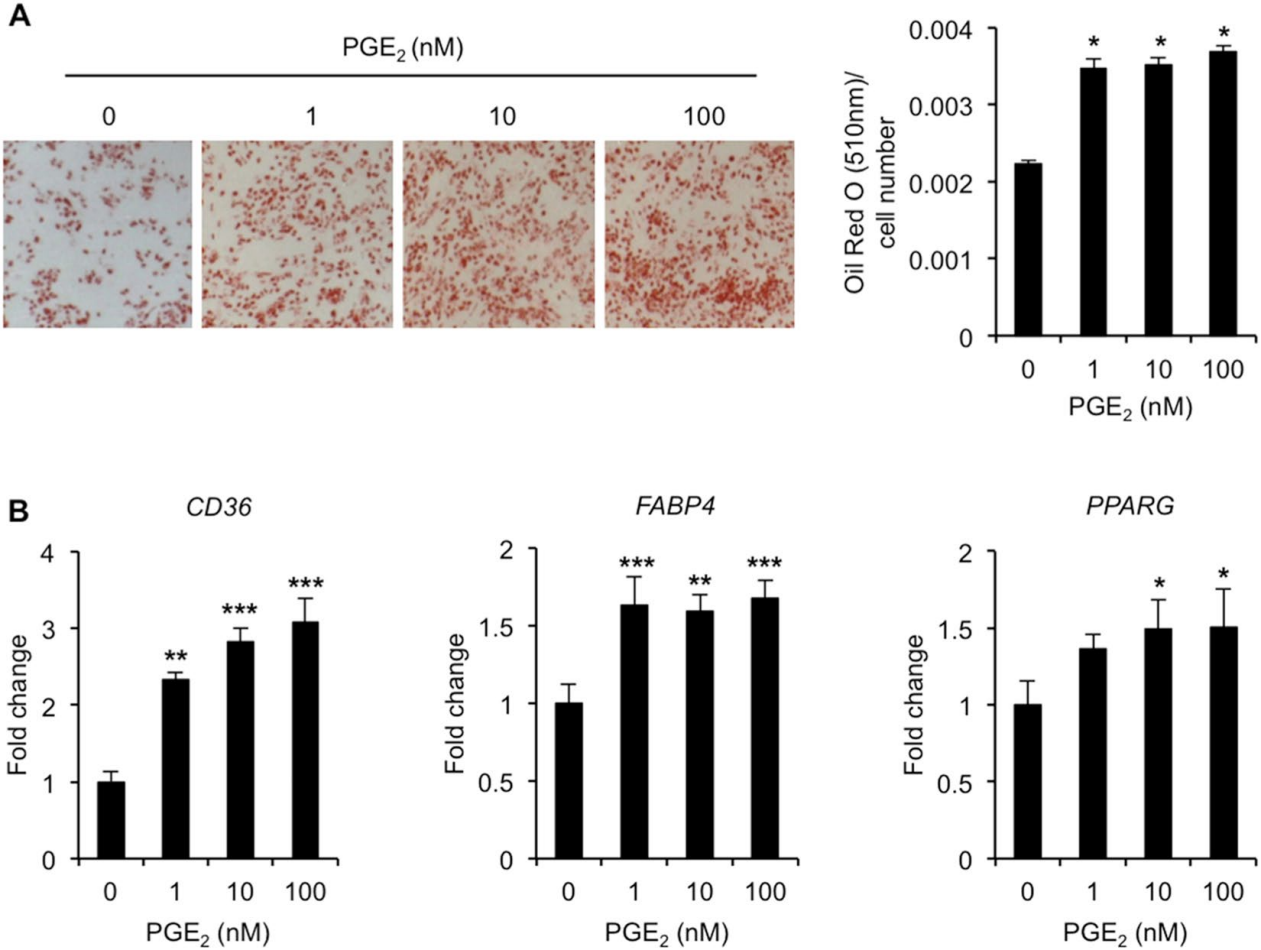
PGE_2_ enhances hBMSC adipogenesis. (**A**) Oil Red O staining was used to assess the influence of continuous PGE_2_ treatment on triglyceride accrual in hBMSC cultures at day 17 post-adipogenic induction. *p < 0.001 as compared to untreated hBMSCs using ANOVA. (**B**) RT-qPCR was used to determine expression levels of adipogenic markers *PPARG*, *FABP4* and *CD36* in hBMSCs at day 17 post-adipogenic induction. Data were normalized to *GUSB* and expressed as fold change as compared to non-induced controls at day 0 (value 1) using the comparative *C*_T_ method. *p < 0.05, **p < 0.01, ***p < 0.001 as compared to untreated hBMSCs using ANOVA. The data represent triplicate determinations and were replicated at least two times. All values are presented as mean ± S.D.

During the course of these studies, we noticed that PGE_2_-treated cells undergoing osteogenesis harbored small numbers of lipid droplet-laden cells (Supplementary Fig. 3A). Furthermore, expression levels of several adipogenic markers were also increased in these cultures (Supplementary Fig. 3B). In order to investigate this further, hBMSCs were cultured under conditions more conducive to adipogenesis, and the effects of PGE_2_ on lipid droplet accrual assessed using Oil Red O staining. In contrast to its inhibitory actions on hBMSC osteogenesis, PGE_2_ treatment had a stimulatory effect on hBMSC adipogenesis as demonstrated by significant increases in Oil Red O staining (Fig. 3A). Furthermore, these effects were accompanied by significant increases in the expression levels of several well-known adipogenic markers including cluster of differentiation 36 (*CD36*), fatty acid binding protein 4 (*FABP4*) and peroxisome proliferator-activated receptor gamma (*PPARG*) (Fig. 3B). These observations therefore indicated that PGE_2_ treatment of hBMSCs not only suppressed their ability to form functional osteoblasts, but also acted to stimulate the formation of lipid laden adipocytes, even under conditions conducive to osteogenesis.

### PGE_2_ mediates its effects through prostaglandin EP2 and EP4 receptors

**Figure 4:**
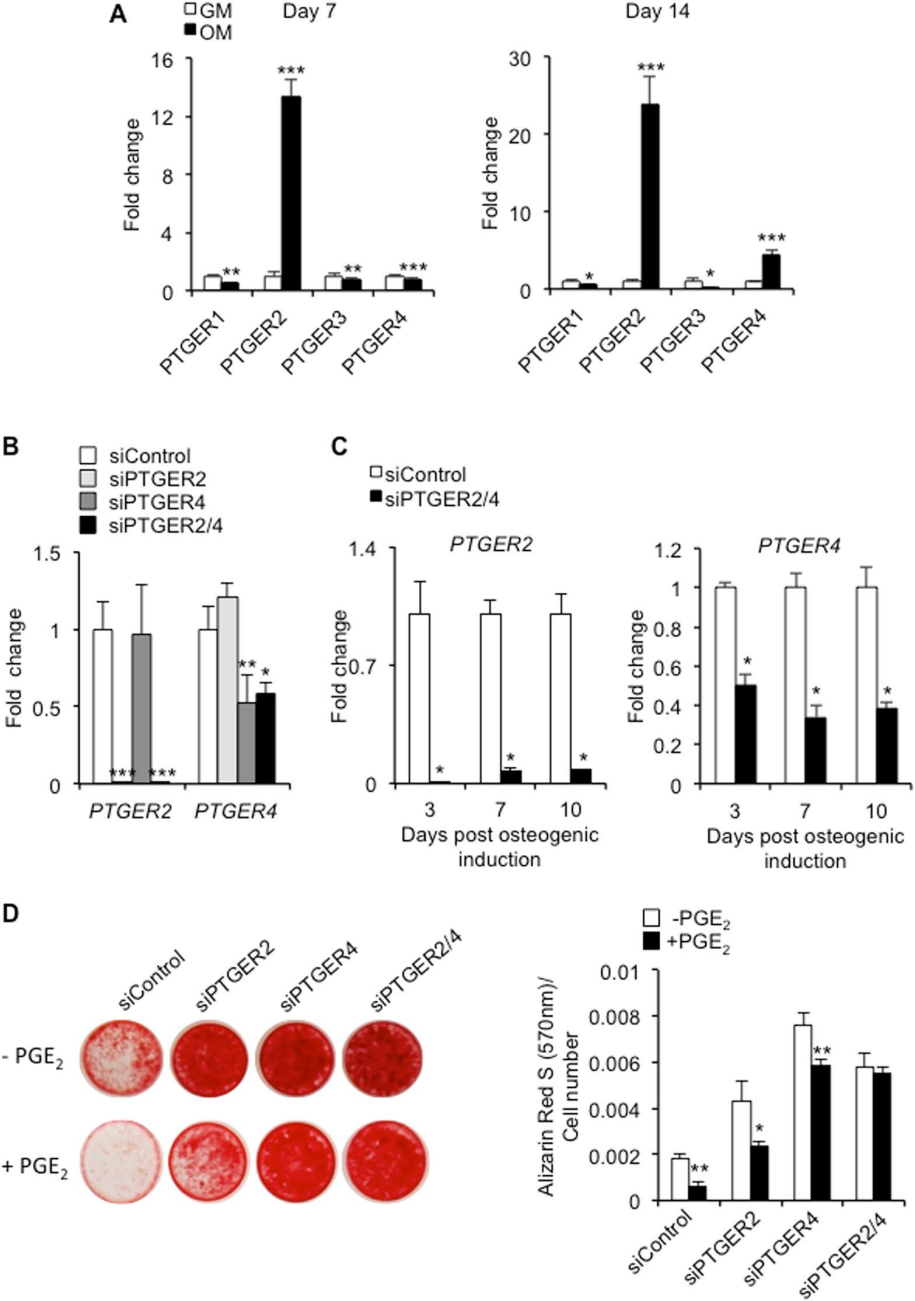
PGE_2_ effects are mediated through specific PGE_2_ receptor subtypes. (**A**) hBMSC were cultured in growth medium (GM) or osteogenic medium (OM), and PGE_2_ receptor gene expression levels determined at day 7 and 14 using RT-qPCR. *p < 0.05, **p < 0.01, ***p < 0.001 as compared to GM using Student’s t-test. (**B**) RT-qPCR was used to assess the short-term effects (48 h) of siRNA specific for *PTGER2* (siPTGER2), *PTGER4* (siPTGER4) or both *PTGER2* and *PTGER4* (siPTGER2/4) on *PTGER2* and *PTGER4* gene expression in hBMSCs undergoing osteogenesis. *p < 0.05, **p < 0.01, ***p < 0.001 as compared to cells treated with scrambled control siRNA (siControl) using ANOVA. (**C**) RT-qPCR was used to assess the long-term effects (3, 7 and 10 days) of siRNA specific for both *PTGER2* and *PTGER4* (siPTGER2/4) on *PTGER2* and *PTGER4* gene expression in hBMSCs undergoing osteogenesis. *p < 0.001 as compared to cells treated with scrambled control siRNA (siControl) using Student’s t-test. (**D**) The effects of continuous PGE_2_ (10 nM) treatment on matrix mineralization in siRNA-treated hBMSCs was assessed at day 15 by Alizarin Red S staining. *p < 0.01, **p < 0.001, as compared to untreated hBMSCs (−PGE_2_) using Student’s t-test. The data represent triplicate determinations and were replicated at least two times. All values are presented as mean ± S.D.

Having identified PGE_2_ as a negative regulator of hBMSC-mediated matrix mineralization, we next sought to identify potential signaling pathways involved in regulating its effects. The responsiveness of cells to prostaglandins is determined by their ability to express specific receptors, and in hBMSCs, PGE_2_ receptors EP2 and EP4 are considered to be the primary targets of PGE_2_^18^. In the current study, expression levels of the gene encoding EP2 (*PTGER2*) were significantly increased in hBMSCs at 7 days (13.3 ± 1.2 fold; *p* < 0.001) and 14 days (23.8 ± 3.6 fold; *p* < 0.001) following osteogenic induction (Fig. 4A). By contrast, expression levels of EP4 (*PTGER4*) were significantly reduced at 7 days (0.8 ± 0.08 fold; *p* < 0.001) following osteogenic induction. However, by day 14, *PTGER4* expression levels were significantly elevated (4.4 ± 0.6 fold; *p* < 0.001), although noticeably reduced in comparison to *PTGER2*. Expression levels of the genes encoding EP1 (*PTGER1*) and EP3 (*PTGER3*) were significantly reduced in hBMSCs exposed to osteogenic induction medium at both time points. Based on these findings, we next performed loss-of-function studies in order to determine the functional roles played by EP2 and EP4 in mediating the inhibitory actions of PGE_2_ on hBMSC-mediated matrix mineralization. We used small interfering RNAs (siRNAs) to specifically suppress the expression of *PTGER2* and/or *PTGER4* in hBMSCs (Fig. 4B), and could demonstrate efficient receptor knockdown for at least 10 days under osteogenic conditions (Fig. 4C). The effects of PGE_2_ on mineral formation were then evaluated after 15 days using Alizarin Red S staining. Suppression of *PTGER2* or *PTGER4* expression resulted in marked increases in Alizarin Red S staining of normally differentiating hBMSCs (Fig. 4D). Moreover, the differences in Alizarin Red S staining between PGE_2_-treated and untreated hBMSCs was reduced from 69% (*p* < 0.001) in siControl-treated cells, to 46% (*p* < 0.01) in siPTGER2-treated cells and 23% (*p* < 0.001) in siPTGER4-treated cells (Fig. 4D). These findings therefore suggested that EP4 may represent the more important of the two PGE_2_ receptors in terms of mediating the actions of PGE_2_ on hBMSCs. However, despite these differences, genetic ablation of both EP2 and EP4 was required to completely alleviate the inhibitory effects of PGE_2_ on hBMSC-mediated matrix mineralization. Similarly, suppression of *PTGER2* and *PTGER4* gene expression also noticeably reduced the capacity for PGE_2_ to enhance lipid droplet accrual in hBMSCs undergoing adipogenesis from 30% (p < 0.001) to 9% (p < 0.05) (Supplementary Fig. 4), indicating their functional role in mediating the effects of PGE_2_ on hBMSC adipogenesis. The possible involvement of additional PGE_2_ receptors, such as EP1 and EP3, was discounted based on the lack of any inhibitory effects of either EP1 or EP3 agonists on hBMSC-derived osteoblast mineralization (Supplementary Fig. 5).

**Figure 5:**
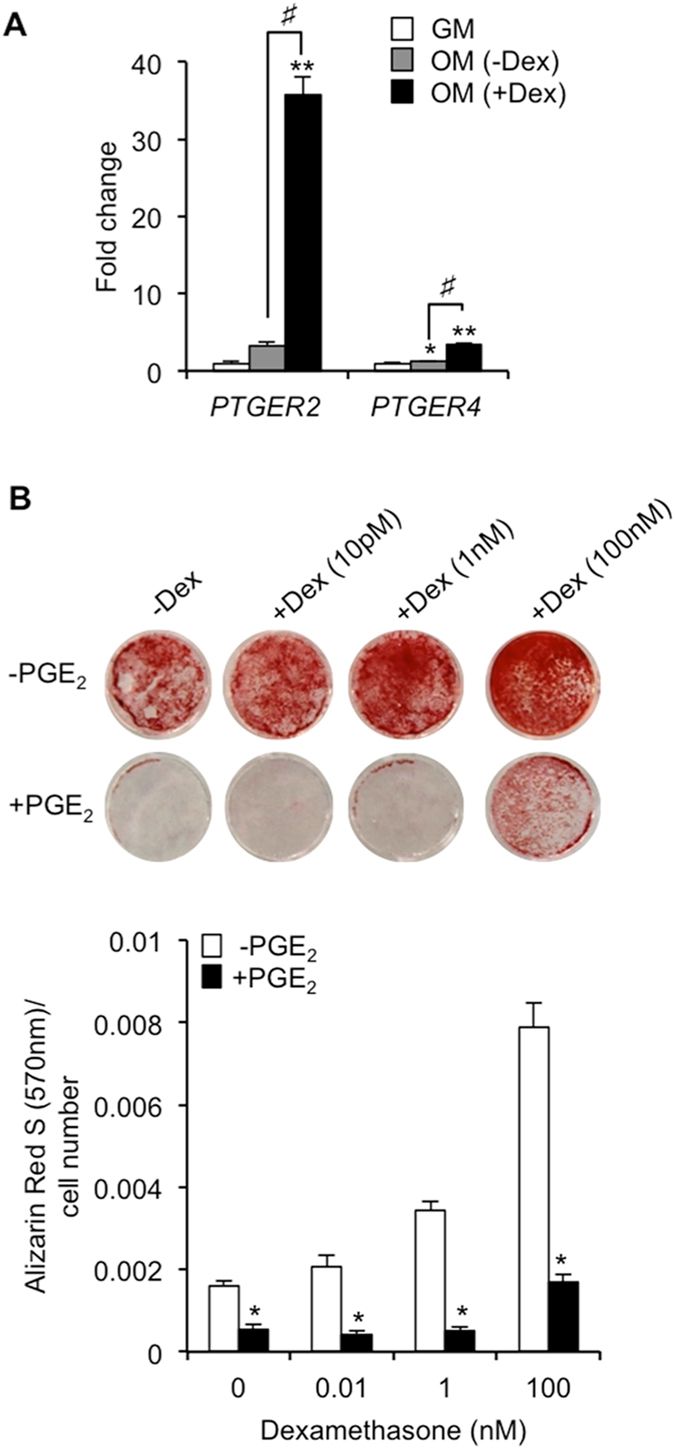
Inhibitory effects of PGE_2_ on hBMSC-mediated matrix mineralization are independent of dexamethasone. (**A**) hBMSC were cultured in growth medium (GM), or osteogenic medium (OM) supplemented with (+Dex) or without (−Dex) dexamethasone, and *PTGER2* and *PTGER4* gene expression levels determined at day 10 using RT-qPCR. *p < 0.05, **p < 0.001 as compared to GM; ^#^ *p* < 0.001 as compared to OM (−Dex) using ANOVA. (**B**) The effect of continuous PGE_2_ (10 nM) treatment on hBMSC-mediated matrix mineralization in the presence or absence of dexamethasone was determined at day 15 by Alizarin Red S staining. *p < 0.001 as compared to untreated hBMSCs (−PGE_2_) using Student’s t-test. The data represent triplicate determinations and were replicated at least two times. All values are presented as mean ± S.D.

It has previously been suggested that dexamethasone present within the culture medium used to induce osteogenesis plays a prominent role in regulating PGE_2_-mediated activation of EP2 and EP4 receptors in hBMSCs^18^. We therefore proceeded to investigate whether alterations in dexamethasone levels could influence PGE_2_ receptor expression and subsequently impact on the efficiency of PGE_2_ to inhibit mineralized matrix formation. Our initial observations identified significant increases in *PTGER2* (35.7 ± 2.3 fold; *p* < 0.001) and, to a lesser extent, *PTGER4* (3.3 ± 0.2 fold; *p* < 0.001) expression in hBMSCs cultured under osteogenic conditions in the presence of dexamethasone (Fig. 5A). As expected, exclusion of dexamethasone from the osteogenic medium impaired hBMSC-derived osteoblast mineralization (Fig. 5B). However, the inhibitory effects of PGE_2_ were not diminished, and reductions in Alizarin Red S staining remained highly significant even when dexamethasone was completely absent (Fig. 5B). These findings therefore indicate that although dexamethasone has the capacity to alter PGE_2_ receptor expression, it does not influence the inhibitory effects of PGE_2_ on hBMSC-mediated matrix mineralization.

### PGE_2_ regulates hBMSC-mediated matrix mineralization via Epac-dependent cAMP signaling

**Figure 6:**
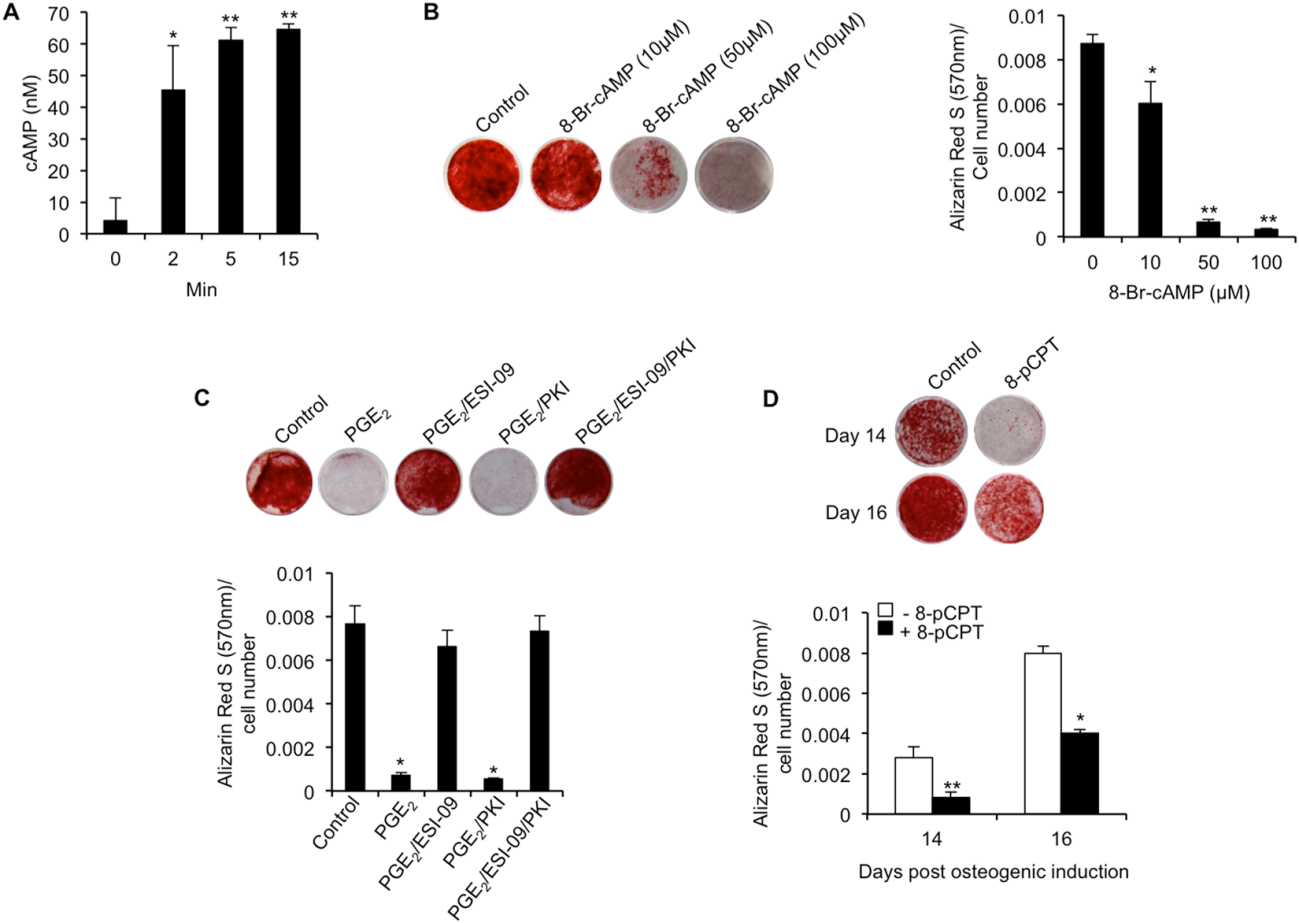
PGE_2_ inhibits hBMSC-mediated matrix mineralization via Epac-dependent cAMP signaling. (**A**) Intracellular cAMP levels were measured in hBMSCs treated with PGE_2_ (100 nM) for 2, 5 and 15 min. *p < 0.01, **p < 0.001, as compared to untreated cells using ANOVA. (**B**) hBMSCs undergoing osteogenic differentiation were treated continuously with cAMP analog 8-Br-cAMP, and matrix mineralization quantified at day 14 by Alizarin Red S staining. *p < 0.01, **p < 0.001 as compared to control using ANOVA. (**C**) hBMSCs were continuously cultured in the absence (control) or presence (PGE_2_) of PGE_2_ (10 nM) with or without Epac inhibitor ESI-09 (10 μM) or PKA inhibitor PKI (10 μM), and matrix mineralization quantified at day 14 by Alizarin Red S staining. *p < 0.001 as compared to control using ANOVA. (**D**) hBMSCs undergoing osteogenic differentiation were treated continuously with cAMP analog 8-pCPT-2-O-Me-cAMP (8-pCPT) (50 μM), and matrix mineralization quantified at day 14 and 16 by Alizarin Red S staining. *p < 0.01, **p < 0.001 as compared to control (−8-pCPT) using Student’s t-test. The data represent triplicate determinations and were replicated at least two times. All values are presented as mean ± S.D.

We next addressed the question of what downstream effectors were activated by PGE_2_-receptor signaling. Prostaglandin EP2 and EP4 receptor signaling is classically regarded as being dependent on intracellular increases in cyclic AMP (cAMP)^22^. Indeed, we observed rapid and significant increases in intracellular cAMP levels in hBMSCs treated with PGE_2_ (Fig. 6A). Furthermore, elevated levels of cAMP were confirmed as having a negative impact on hBMSC-mediated matrix mineralization as evidenced by significant reductions in Alizarin Red S staining in hBMSC cultures treated with increasing concentrations of the cAMP analog 8-Br-cAMP (Fig. 6B). We next sought to establish the downstream signaling events responsible for mediating the cAMP response. Protein kinase A (PKA) and exchange protein directly activated by cAMP (Epac) are two of the most well studied downstream effectors of cAMP, and have been implicated in human multipotent stromal cell (hMSC) lineage commitment^23, 24^. Pharmacological inhibition of Epac using the specific Epac1 inhibitor ESI-09^25^ resulted in complete rescue of matrix mineralization in hBMSC cultures treated with PGE_2_ (Fig. 6C). By contrast, the PKA inhibitor peptide PKI^26^ failed to significantly alleviate the inhibitory effects of PGE_2_. Interestingly, although hBMSC-mediated matrix mineralization in the absence of PGE_2_ was not significantly affected by ESI-09, it was significantly enhanced by PKI (Supplementary Fig. 6). In order to confirm that activation of Epac alone was able to simulate the inhibitory effects of PGE_2_, we treated hBMSCs with the Epac-specific cAMP analog 8-pCPT-2-O-Me-cAMP, and assessed its ability to alter Alizarin Red S staining. Indeed, we found that 8-pCPT-2-O-Me-cAMP could significantly inhibit matrix mineralization at concentrations equivalent to the non-selective cAMP analog 8-Br-cAMP (Fig. 6D).

**Figure 7:**
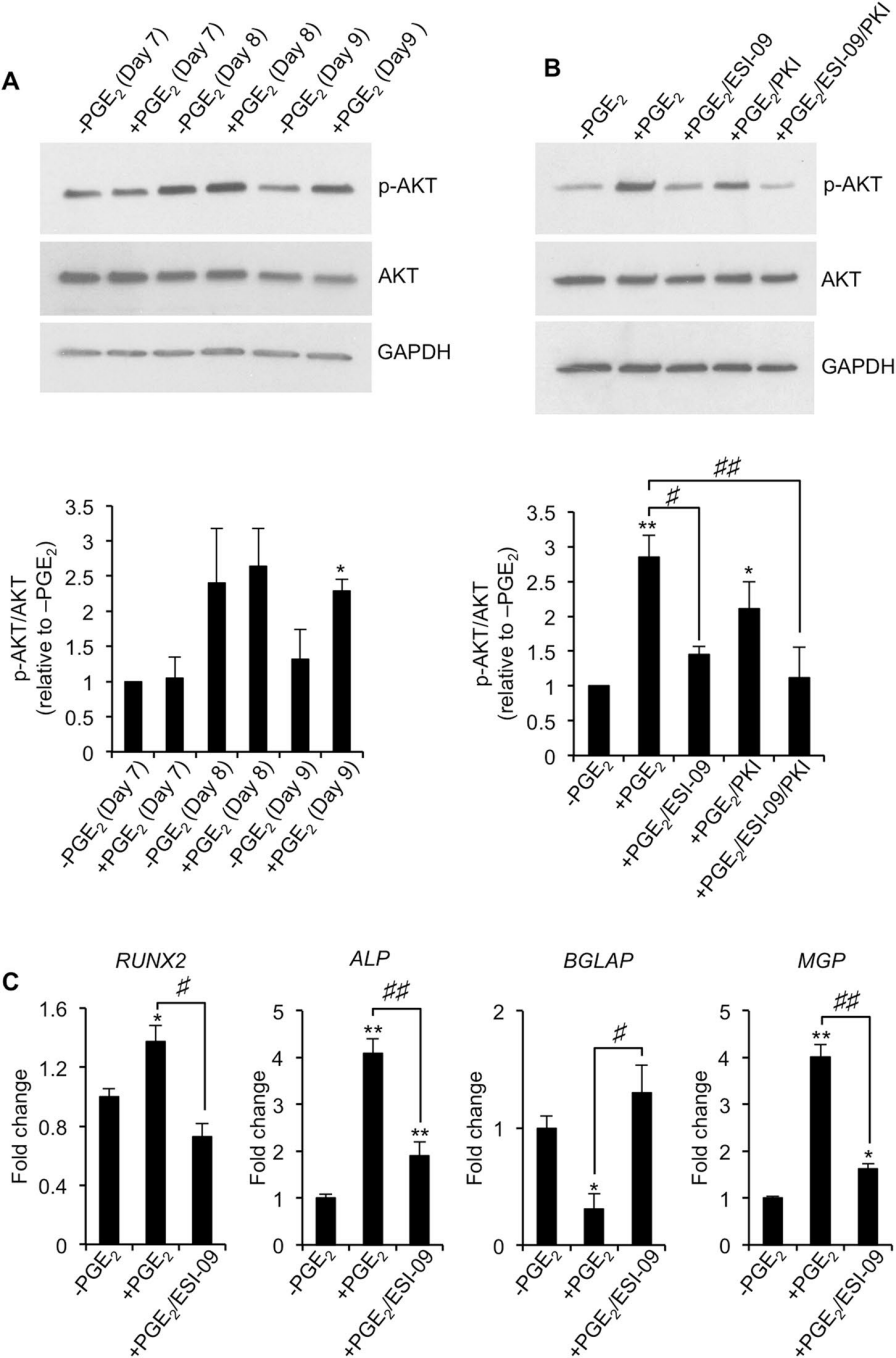
PGE_2_ activates AKT in an Epac-dependent manner. (**A**) hBMSCs were cultured continuously in the absence (−PGE_2_) or presence (+PGE_2_) of PGE_2_ (10 nM), and AKT phosphorylation levels determined by Western blot analysis at day 7, 8 and 9. *p < 0.01 as compared to −PGE_2_ using Student’s t-test. (**B**) hBMSCs were cultured continuously in the absence (−PGE_2_) or presence (+PGE_2_) of PGE_2_ (10 nM) with or without Epac inhibitor ESI-09 (10 μM) or PKA inhibitor PKI (10 μM), and AKT phosphorylation levels determined by Western blot analysis at day 9. *p < 0.01, **p < 0.001 as compared to -PGE_2_ using ANOVA. ^#^ *p* < 0.01, ^##^ *p* < 0.001 using ANOVA. In both cases, GAPDH served as a loading control and representative cropped blots shown. (**C**) hBMSCs were cultured continuously in the absence (−PGE_2_) or presence (+PGE_2_) of PGE_2_ (10 nM) with or without Epac inhibitor ESI-09 (10 μM), and *RUNX2*, *ALP*, *BGLAP* and *MGP* expression levels determined by RT-qPCR at day 17. *p < 0.05, **p < 0.001 as compared to -PGE_2_ using ANOVA. ^#^ *p* < 0.01, ^##^ *p* < 0.001 using ANOVA. The data represent triplicate determinations and were replicated at least two times. All values are presented as mean ± S.D.

It was previously shown that Epac activation reduced hBMSC-mediated matrix mineralization independently of PKA, and that this was associated with increased AKT phosphorylation^23^. We therefore reasoned that if Epac, and not PKA, was responsible for mediating the inhibitory actions of PGE_2_ on hBMSC-mediated matrix mineralization, then alterations in AKT activity should accompany these changes. In order to investigate this, hBMSCs undergoing osteogenesis were treated with PGE_2_ for 7 days to ensure an adequate cellular response based on our previous findings (Fig. 2), and AKT activation assessed by immunoblotting after 24 h (day 8) and 48 h (day 9) following media change and re-addition of PGE_2_. Phosphorylated AKT levels were comparable between untreated and PGE_2_-treated hBMSCs after 7 days of osteogenic differentiation, and continued to remain so for the next 24 h following media replenishment (Fig. 7A). However, after 48 h, phosphorylated AKT levels were significantly elevated in PGE_2_-treated hBMSCs as compared to untreated hBMSCs. These findings indicated that PGE_2_ treatment of hBMSCs resulted in sustained AKT activation, and thereby provided a possible mechanism to account for its effects on hBMSC-mediated matrix mineralization. In order to confirm Epac as the primary mediator of PGE_2_’s effects on AKT activation, we measured the level of AKT activation in PGE_2_-treated hBMSCs cultured in the presence of the Epac inhibitor ESI-09. Indeed, PGE_2_’s stimulatory effects on AKT phosphorylation were significantly diminished in hBMSCs treated with ESI-09 (Fig. 7B). By contrast, inhibition of PKA in PGE_2_-treated hBMSCs failed to significantly alter AKT phosphorylation levels as compared to hBMSCs treated with PGE_2_ alone. Finally, we asked the question whether inhibition of Epac activation could also influence the effects of PGE_2_ on osteogenic gene expression, and thereby provide a possible molecular mechanism through which PGE_2_ regulates hBMSC-derived osteoblast function. Indeed, the ability of PGE_2_ to induce changes in the expression patterns of *RUNX2*, *ALP*, *BGLAP* and *MGP* was almost completely prevented by Epac inhibitor ESI-09 (Fig. 7C).

## Discussion

The importance of PGE_2_ in bone formation has been confirmed under physiological and pathological conditions using experimental animal models in which PGE_2_^6–8^, or PGE_2_ receptor agonists have been administered^27, 28^, or where specific enzymes responsible for PGE_2_ production have been deleted^29, 30^. The pro-osteogenic effect of PGE_2_ has been further substantiated by findings from numerous *in vitro* studies using murine- or rat-derived BMSCs and osteoblasts^13–15, 31–33^. However, only a limited number of investigations have been performed into the effects of PGE_2_ on human bone formation, the results of which raise concerns about the translational value of using small animal models to evaluate the effects of PGE_2_ on bone metabolism. An early study by Evans *et al*.^34^ demonstrated that PGE_2_ could significantly inhibit osteocalcin production by human osteoblasts at concentrations as low as 1 nM^34^. Equivalent concentrations of PGE_2_ were also shown to significantly impair ALP activity in human MG63 osteoblast-like cells^35^. More recently, PGE_2_ was shown to effectively suppress hBMSC-mediated matrix mineralization^18^. Our new findings presented here support the concept that continuous treatment with PGE_2_ acts to impair matrix mineralization by hBMSCs committed toward osteoblasts. Accordingly, the osteoblast-specific marker osteocalcin was significantly reduced in late-stage cultures (day 17) treated with PGE_2_. Furthermore, we also demonstrated that PGE_2_ treatment significantly increased matrix gla protein (*MGP*) expression levels. MGP is a potent inhibitor of matrix mineralization^36^, and increases in its production may have certainly contributed to the observed reductions in Alizarin Red S staining. However, these findings are somewhat confounded by the fact that PGE_2_ treatment actually enhanced osteogenic markers *RUNX2* and *ALP* in hBMSCs at the early stages of osteogenic differentiation (day 3 and 7) through to the osteoblast maturation phase (day 17). At first sight, these results would suggest that the inhibitory effects of PGE_2_ on hBMSC-mediated matrix mineralization may simply be due to the fact that hBMSCs were continuously exposed to PGE_2_, and that its removal during the early stages of hBMSC differentiation could alleviate these effects and possibly even promote osteogenesis. However, we saw no evidence of enhanced matrix mineralization following the removal of PGE_2_ from the culture system at various time points during the first 7 days of hBMSC osteogenesis. Moreover, exposure of hBMSCs to PGE_2_ for 7 days only, still led to significant reductions in matrix mineralization. Although regarded as being of critical importance during the early stages of hBMSC osteogenesis, *RUNX2* actually imparts a negative influence on osteoblast maturation and subsequent bone formation^37^. Furthermore, expression levels of *BGLAP* are noticeably reduced in the bones from transgenic mice overexpressing *RUNX2* as compared to wild-type littermates^37^. It’s possible therefore that the sustained increases in *RUNX2* expression following PGE_2_ treatment may have had a detrimental effect on osteoblast maturation, thereby resulting in deficiencies in hBMSC-mediated matrix mineralization.

In addition to its inhibitory actions on hBMSC-mediated matrix mineralization, PGE_2_ also has the capacity to enhance hBMSC adipogenesis^18^. Indeed, not only were we able to confirm this, but we also demonstrated that PGE_2_ treatment promoted an adipogenic phenotype in hBMSCs even under pro-osteogenic culture conditions. As is the case with osteogenesis, investigations into the role of PGE_2_ in adipogenesis have primarily be conducted in small animal species, and contrary to its effects on hBMSCs, have identified PGE_2_ as a negative regulator of adipogenesis^38–40^. Our findings therefore provide important additional insights into how PGE_2_ may act to influence human bone formation, where increases in BMSC adipogenesis at the expense of osteogenesis would be expected to impart a negative influence on bone quality^41^. Although no studies have yet directly investigated the role of PGE_2_ in human bone metabolism, some initial insights have been gleaned from bone mineral density (BMD) measurements performed on patients treated with nonsteroidal anti-inflammatory drugs (NSAIDs) targeting COX-1 or COX-2. In this regard, several clinical studies have shown that inhibition of prostaglandin production by daily treatments of NSAIDs had a positive influence on BMD in elderly men and/or women^42–44^. However, these findings are confounded by those from a more recent study in which the treatment of elderly men and women with COX-2 inhibitors reportedly led to an overall decrease in BMD in men, whilst enhancing BMD in women^45^. Although the reason for these discrepancies remains unclear, it was hypothesized that the anti-inflammatory properties of COX-2 inhibitor treatment most likely contributed to the improved BMD in postmenopausal women. The ability of NSAIDs to reduce the inflammatory response may also account for their apparent effects on bone healing in humans, where in the majority of cases, higher incidences of non-unions have been reported in patients treated with NSAIDs^46^. Clearly, more in-depth studies are needed to ascertain whether the effects of prostaglandin inhibition on bone formation are in any way related to alterations in BMSC activity. One approach may be to compare the osteogenic potential of BMSCs harvested from NSAID-treated patients with those from untreated patients, a strategy previously used to demonstrate the osteoinductive effects of bisphosphonates on hBMSCs^47^.

The effects of PGE_2_ of hBMSC-mediated matrix mineralization are thought to be mediated primarily through PGE_2_ receptors EP2 and EP4^18^. We demonstrated that genes encoding EP2 (*PTGER2*) and EP4 (*PTGER4*) were selectively upregulated in hBMSCs at later stages of osteogenesis. By comparison, the expression levels of the genes encoding EP1 (*PTGER1*) and EP3 (*PTGER3*) were actually downregulated in response to osteogenic induction, and therefore most likely account for the minimal effects observed on hBMSC osteogenesis following treatment with EP1 and EP3 agonists. However, despite identifying dexamethasone as a potent stimulator of *PTGER2* expression, and, to a lesser extent, *PTGER4* expression, the response of hBMSCs to PGE_2_ was independent of dexamethasone. The fact that dexamethasone’s ability to enhance *PTGER4* expression was markedly diminished in comparison to that of *PTGER2*, would suggest that PGE_2_-mediated inhibition of hBMSC-mediated matrix mineralization was regulated mainly via PGE_2_ receptor subtype EP4. Certainly, treatment of hBMSCs with *PTGER4* siRNA proved more effective than *PTGER2* siRNA in rescuing matrix mineralization by hBMSCs treated with PGE_2_, although downregulation of both *PTGER2* and *PTGER4* gene expression was required for complete rescue. It is also interesting to note that the mere action of silencing *PTGER2* or *PTGER4* expression resulted in marked increases in matrix mineralization, suggesting that endogenous PGE_2_ also plays a role in directing hBMSC-mediated matrix mineralization.

Despite our current knowledge of PGE_2_ receptor signaling pathways, the downstream events responsible for mediating the effects of PGE_2_ on hBMSC-mediated matrix mineralization remain undetermined. Here we have demonstrated that PGE_2_ increased cAMP levels in hBMSCs, and that Epac was indispensible for its inhibitory actions on mineralized matrix formation. These findings are in agreement with those previously reported by Tang *et al*.^23^, whereby the Epac-activating cAMP analog 8-pCPT-2-O-Me-cAMP effectively inhibited hBMSC-mediated matrix mineralization^23^. In addition to identifying Epac as the primary signaling component required for PGE_2_ to elicit its inhibitory action on hBMSC-mediated matrix mineralization, we also demonstrated it as being of major importance in PGE_2_-induced AKT activation. The ability of Epac to activate AKT in response to increases in intracellular cAMP levels has previously been shown in both hBMSCs^23^ and multipotent stromal cells derived from human umbilical cord blood (hUCB-MSCs)^24^. Moreover, the observed increases in AKT phosphorylation were demonstrated as being independent of PKA activity. Similarly, in our study, PKA inhibition failed to significantly influence the effects of PGE_2_ on AKT phosphorylation in hBMSCs undergoing osteogenesis. This provided further confirmation that Epac, rather than PKA, was the main cAMP effector involved in mediating the effects of PGE_2_ on hBMSC-mediated matrix mineralization. The AKT signaling pathway is well regarded as being an important contributor to osteogenesis as revealed by studies using mice deficient in *Akt1* and *Akt2* ^48, 49^. Therefore, it may seem counter intuitive that increases in AKT activity could impede hBMSC osteogenesis. However, more recent studies have since demonstrated that loss of *Akt1* leads to enhanced osteogenic differentiation of mouse bone progenitor cells from a variety of different sources^50^. It’s also interesting to note that RUNX2 transcriptional activity is positively regulated by AKT^51^, and its gene expression can be induced in tumour cells in which AKT is constitutively activated^52^. RUNX2 may therefore represent a downstream effector of sustained AKT activation, and a possible mediator through which PGE_2_ enforces its detrimental actions on hBMSC-derived osteoblast maturation and matrix mineralization. This concept is supported by our finding that restoration of AKT activation following inhibition of Epac also alleviated the effects of PGE_2_ on the expression of *RUNX2* and its downstream effector genes *ALP*, *BGLAP* and *MGP*. Therefore, it is tempting to speculate that the inhibitory actions of PGE_2_ on matrix mineralization are reliant on AKT-mediated induction of RUNX2, leading to alterations in the production of key regulators of osteoblast differentiation and maturation. Additionally, AKT activation is also an integral part of adipogenesis, being regulated both temporally and spatially^53^. The sustained activation of AKT observed in PGE_2_-treated hBMSCs may have therefore also served to promote adipogenesis, at the expense of osteogenesis. Clearly, more in-depth studies are needed to clarify the role of AKT signaling in mediating the effects of PGE_2_ on hBMSC osteogenesis and adipogenesis.

**Figure 8:**
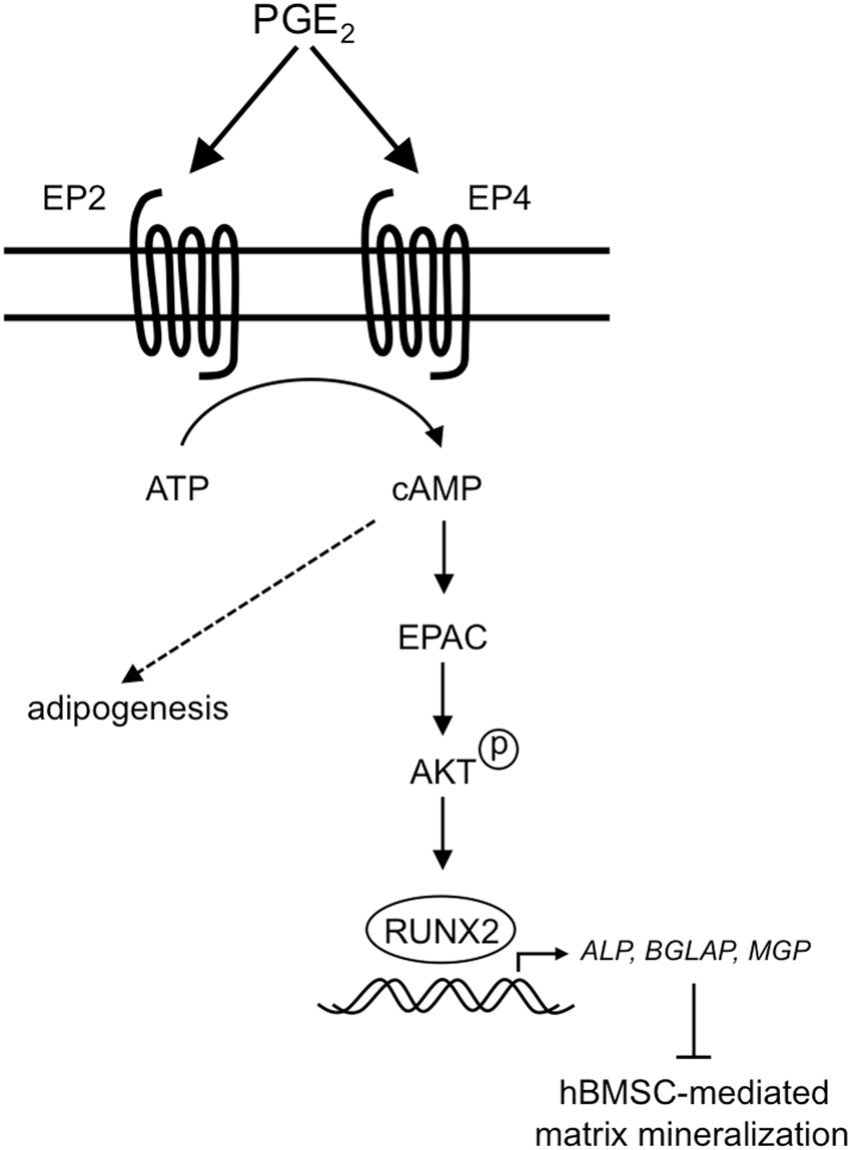
Proposed mechanism by which PGE_2_ exerts its influence over hBMSC-mediated matrix mineralization. Based on the findings presented in the current report, we propose that PGE_2_ increases intracellular cAMP levels via receptors EP2 and EP4, leading to activation of Epac, which in turn acts to sustain AKT phosphorylation levels. Prolonged AKT activation ultimately results in impaired hBMSC-derived osteoblast maturation and matrix mineralization, possibly by inducing temporal changes in the production and activity of *RUNX2* and its downstream target genes (e.g. *ALP*, *BGLAP* and *MGP*). The signaling pathways responsible for mediating the stimulatory effects of PGE_2_ on hBMSC adipogenesis still remain to be determined.

In conclusion, our results demonstrate that the inhibitory actions of PGE_2_ on hBMSC-mediated matrix mineralization involve EP2 and EP4 signaling, and are reliant on the cAMP-Epac pathway. Furthermore, our data also implicate AKT as a downstream effector of PGE_2_-Epac signaling, where it most likely acts to disrupt the temporal expression of genes critically involved in the differentiation and maturation of hBMSC-derived osteoblasts (Fig. 8). Additional studies are underway to determine whether similar signaling events are also responsible for regulating the stimulatory effects of PGE_2_ on hBMSC adipogenesis. Taken together, these findings provide important insights into the signaling events controlling hBMSC lineage commitment and as such, may help in deciphering the role played by PGE_2_ signaling in pathological conditions such as osteoporosis, where dysregulation of BMSC differentiation and BMSC-mediated matrix mineralization is an underlying feature. Furthermore, our data underline the differential effects of PGE_2_ on MSC differentiation between species, and imply that some caution may be warranted when translating results from animal studies to the clinic.

## Materials and Methods

### Materials

Primary antibodies against AKT and p-AKT (Ser473) were purchased from Cell Signaling Technology (Leiden, The Netherlands). Anti-GAPDH was from LabForce (Muttenz, Switzerland). HRP-labeled secondary antibodies were purchased from Jackson ImmunoResearch (Suffolk, UK). Prostaglandins E_2_ and D_2_, EP1 agonist (17-Phenyl-trinor-prostaglandin E2), EP3 agonist (Sulprostone), cAMP analogs 8-pCPT-2′-O-Me-cAMP and 8-Br-cAMP, and PKI 14–22 were all purchased from Enzo Life Science (Lausen, Switzerland). The Epac inhibitor (ESI-09) was purchased from Sigma-Aldrich (Buchs, Switzerland).

### Human bone marrow stromal cell (hBMSC) culture

All experiments were performed using human BMSCs purchased from Lonza (Verviers, Belgium). In some instances, BMSCs from the bone marrow of additional human donors undergoing routine surgical procedures were also used to confirm the reproducibility of the test system. The harvesting of human material was performed in accordance with the relevant guidelines and regulations following informed patient consent and approval by the ethics commission for the Canton of Zurich, and BMSCs isolated and purified using previously established protocols^54^. Cell cultures were maintained at 37 °C, in 5% CO_2_ and 98% humidity in normal growth medium consisting of Dulbecco’s modified eagle medium (DMEM-low glucose, with GlutaMAX) (Thermo Fisher Scientific, Reinach, Switzerland), supplemented with 10% fetal bovine serum (FBS) (Sigma-Aldrich), penicillin/streptomycin (50 units/ml; 50 μg/ml). Cells were used between passage 5 and 8^55^.

### Osteogenic differentiation of hBMSCs

hBMSCs were seeded at a starting density of 10,000–15,000 cells/cm^2^, cultured overnight in normal growth medium, and then induced to undergo osteogenesis for up to 21 days in normal growth medium supplemented with 100 nM dexamethasone, 10 mM β-glycerophosphate and 50 µM L-Ascorbic acid 2-phosphate (all from Sigma-Aldrich) unless otherwise stated^56^. Matrix mineralization by hBMSC-derived osteoblasts was determined at specified time points using Alizarin Red S (Sigma-Aldrich), and staining quantified by measuring the optical densities of extracted stain at 570 nm. Differences in Alizarin Red S staining due to cell proliferation were accounted for by normalization of optical densities to cell number as previously described^56^.

### Adipogenic differentiation of hBMSCs

hBMSCs were seeded at a starting density of 24,000 cells/cm^2^, cultured overnight in normal growth medium, and then induced to undergo adipogenesis for up to 17 days using DMEM-high glucose (with GlutaMAX), supplemented with 10% FBS, 1 μM dexamethasone, 10 μg/ml insulin, 0.1 mM Indomethacin, and 0.5 mM isobutylmethylxanthine (IBMX) (all from Sigma-Aldrich). Cells were exposed to adipogenic induction medium for 3 days and subsequently maintained in IBMX-free adipogenic induction medium thereafter^55^. Triglyceride accumulation in hBMSCs undergoing adipogenesis was identified at specified time points using Oil Red O (Sigma-Aldrich), and staining quantified by measuring the optical densities of extracted stain at 510 nm. Differences in Oil Red O staining due to cell proliferation were accounted for by normalization of optical densities to cell number as previously^55^.

### hBMSC treatment

hBMSCs undergoing osteogenic or adipogenic differentiation were treated with prostaglandin D_2_ or E_2_, prostaglandin EP1 or EP3 receptor agonists, cAMP analogs 8-pCPT-2-O-Me-cAMP or 8-Br-cAMP, or vehicle control for different time periods at the concentrations indicated. Cell culture media containing the specified treatment agents was regularly replenished every 3 to 4 days throughout the course of the experiment. Where stated, cells were also pre-treated with pharmacological or peptide inhibitors targeting specific signaling pathways 1 h prior to prostaglandin treatment. In order to investigate whether the effects of PGE_2_ on hBMSC-mediated matrix mineralization were dependent on the osteogenic differentiation stage of hBMSCs, PGE_2_ was added to cells at different time points following osteogenic induction. The effect of duration of exposure to PGE_2_ was assessed by altering the number of times media was replenished with fresh PGE_2_ during the course of hBMSC osteogenesis.

### Gene expression analysis

Gene expression levels of osteogenic or adipogenic markers were quantified by RT-qPCR using TaqMan Gene Expression Assays (Thermo Fisher Scientific) (Supplementary Table S1) as previously described^55^. Total RNA was harvested from cells at selected time points during differentiation and 0.5 μg of total RNA reverse-transcribed using Superscript II (Thermo Fisher Scientific). An equivalent of 10 ng total RNA was applied as cDNA template in the successive RT-qPCR reaction using the StepOnePlus (Thermo Fisher Scientific). Values were normalized to *GUSB* and presented as fold change according to the 2^−∆∆*C*T^ method.

### Small Interfering RNA (siRNA) Studies

Gene knockdowns were performed with Silencer Select siRNA oligos (Thermo Fisher Scientific) specific for *PTGER2* (s11449) or *PTGER4* (s60395), or combinations thereof, using the NEON transfection method (Thermo Fisher Scientific) as previously described^55^. Briefly, hBMSCs (1 × 10^5^ cells) were transfected with up to 20 nM of siRNAs or negative control siRNA (Negative Control-1), and seeded in cell culture plates with fresh growth medium (without antibiotics) for 24 h at 37 °C, 5% CO_2_. Medium was then replaced with osteogenic induction medium, and knockdown efficiency confirmed at selected time points by RT-qPCR.

### Immunoblotting

hBMSCs were induced to undergo osteogenesis in the presence or absence of PGE_2_ (10 nM), ESI-09 (10 μM) or PKI 14–22 (10 μM), and cells lysed at day 7, 8 and 9 using CelLytic M (Sigma-Aldrich) supplemented with protease and phosphatase inhibitor cocktails (Sigma-Aldrich). Protein concentrations were determined by Bradford-based protein assay (Bio-Rad). Protein samples (20 μg) were boiled for 5 min in loading buffer (50 mM Tris-HCl, pH 6.8, 2% (v/v) SDS, 10% (v/v) glycerol, 100 mM DTT, 0.002% (w/v) bromophenol blue) and subjected to SDS-PAGE using 4–15% precast Tris-HCl gels (BioRad). Protein was then electroblotted onto PVDF membranes using the Trans-Blot Turbo blotting system (BioRad). Membranes were subsequently blocked with 5% (w/v) skim milk in TBST (50 mM Tris-HCl, pH 7.6, 150 mM NaCl, 0.05% (v/v) Tween 20) for 1 h at room temperature, and then incubated with primary antibodies against AKT, p-AKT, or GAPDH overnight at 4 °C at the recommended dilutions in blocking buffer. Antibody binding was detected using HRP-conjugated secondary antibodies followed by incubation in Super Signal West Pico Chemiluminescent Substrate (Life Technologies) and exposed to x-ray film. The same protein samples were run on three separate gels and protein levels quantified using NIH ImageJ software. Phosphorylated and non-phosphorylated protein values were first normalized to GAPDH loading control and then the phosphorylation to total protein ratio calculated using the normalized values.

### cAMP assay

hBMSCs were seeded at 15,000 cells/cm^2^ in 96-well plates, and induced to undergo osteogenesis for 3 days. Medium was then replaced with PBS containing PGE_2_ (100 nM) for up to 15 min. Measurement of intracellular cAMP levels in hBMSCs was then performed using the cAMP-Glo Kit (Promega, Dübendorf, Switzerland) according to the manufacturer’s protocol. The resulting luminescence was measured using a multiplate reader and cAMP concentration calculated according to manufacturer’s protocol (Tecan, Männedorf, Switzerland).

### Statistical Analysis

Statistical significance was determined by Student’s t test for comparison of two groups and one-way analysis of variance (ANOVA) with Tukey’s post hoc test for multiple group comparisons. In all cases, a *p*-value of <0.05 was considered statistically significant, and all data were expressed as mean ± standard deviation (S.D).

## Electronic supplementary material

Supplementary Information
