# Supplementary material for: Prostaglandin E2 inhibits matrix mineralization by human bone marrow stromal cell-derived osteoblasts via Epac-dependent cAMP signaling
Source: Sci Rep. 2017 May 22;7:2243. doi: 10.1038/s41598-017-02650-y (PMC5440379; doi:10.1038/s41598-017-02650-y)
Supplement: Supplementary file 1 — Supplementary Information [file 41598_2017_2650_MOESM1_ESM.pdf]

Prostaglandin E_2 inhibits matrix mineralization by human bone marrow stromal cell-derived osteoblasts via Epac-dependent cAMP signaling

Ali Mirsaidi[a¶], André N. Tiaden[a¶], Peter J. Richards[a,b*]

[a]Bone and Stem Cell Research Group, CABMM, University of Zurich, 8057 Zurich, Switzerland. [b]Zurich Center for Integrative Human Physiology (ZIHP), University of Zurich, 8057 Zurich, Switzerland.

Supplementary Table S1. List of TaqMan Gene Expression Assays used in RT-qPCR

Gene Symbol	Protein Product	Assay ID [a]
BGLAP	Bone gamma-carboxyglutamate (gla) protein	Hs01587814_g1
MGP	Matrix gla protein	Hs00179899_m1
SPP1	Secreted phosphoprotein 1	Hs00959010_m1
CD36	Cluster of Differentiation 36	Hs01567185_m1
SP7	Osterix	Hs00541729_m1
RUNX2	Runt-related transcription factor 2	Hs01047976_m1
ALPL	Alkaline phosphatase	Hs00758162_m1
SOST	Sclerostin	Hs00228830_m1
DMP1	Dentin matrix acidic phosphoprotein 1	Hs01009391_g1
FABP4	Fatty acid binding protein 4	Hs00173720_m1
PPARG	Peroxisome proliferator activated receptor γ	Hs01115513_m1
PTGER1	Prostaglandin E receptor 1	Hs00909194_g1
PTGER2	Prostaglandin E receptor 2	Hs04183523_m1
PTGER3	Prostaglandin E receptor 3	Hs00168755_m1
PTGER4	Prostaglandin E receptor 4	Hs00168761_m1
GUSB	Glucuronidase, beta	Hs99999908_m1

[a]TaqMan Expression Assay identity code according to supplier (Thermo Fisher Scientific, Reinach, Switzerland).

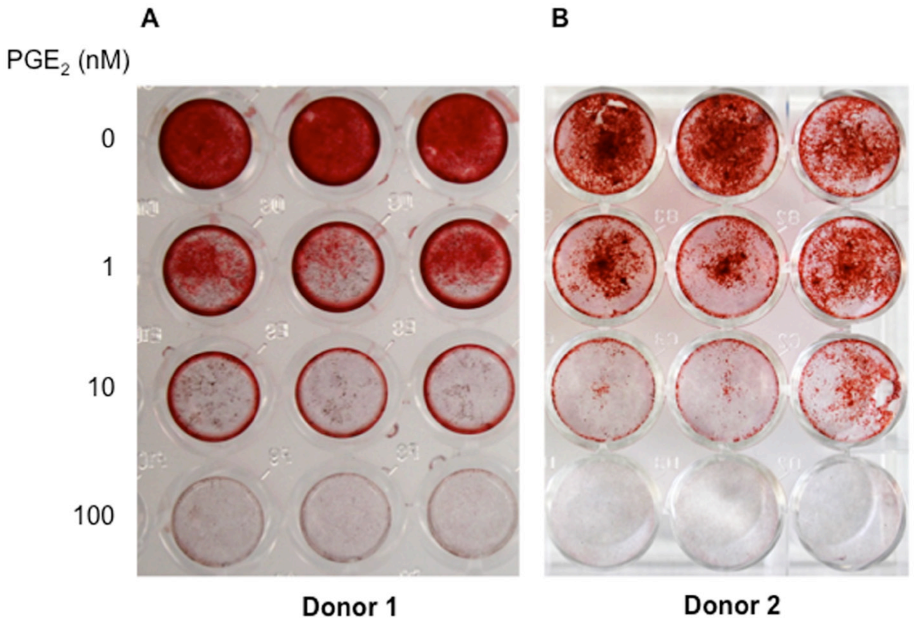

Supplementary Figure S1. PGE_2 inhibits hBMSC-mediated matrix mineralization. Alizarin Red S staining was used to assess the influence of continuous PGE_2 treatment at varying concentrations on matrix mineralization in hBMSC cultures. BMSCs were harvested from two different donors and Alizarin Red S staining performed at day 14 (A) or day 21 (B) post-osteogenic induction.

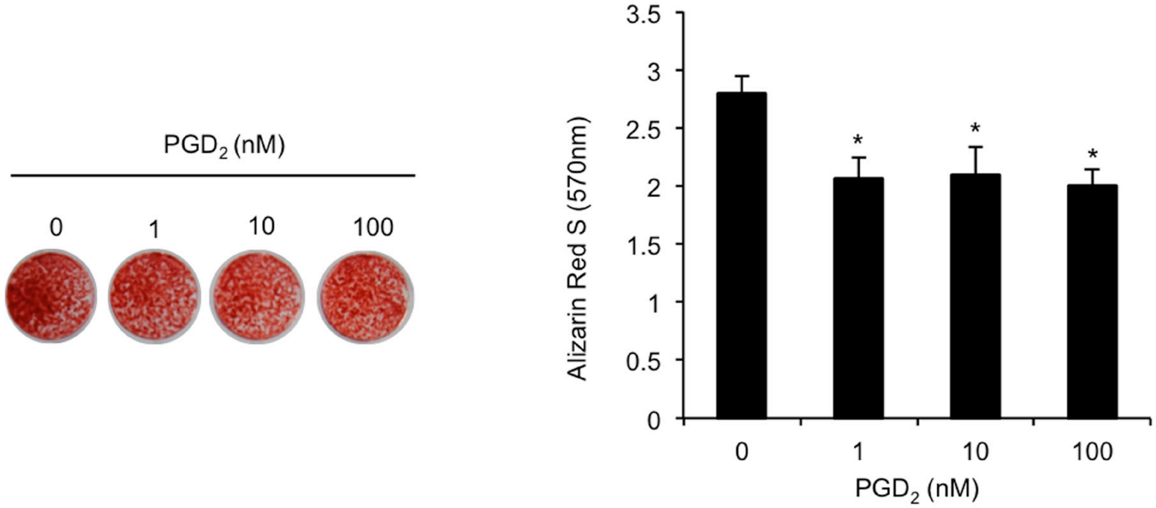

Supplementary Figure S2. Influence of PGD_2 on hBMSC-mediated matrix mineralization. Alizarin Red S staining was used to assess the influence of continuous PGD_2 treatment on matrix mineralization in hBMSC cultures at day 14 post-osteogenic induction. * $p < 0.01$ as compared to untreated hBMSCs using ANOVA. The data represent triplicate determinations and were replicated at least two times. All values are presented as mean ± S.D.

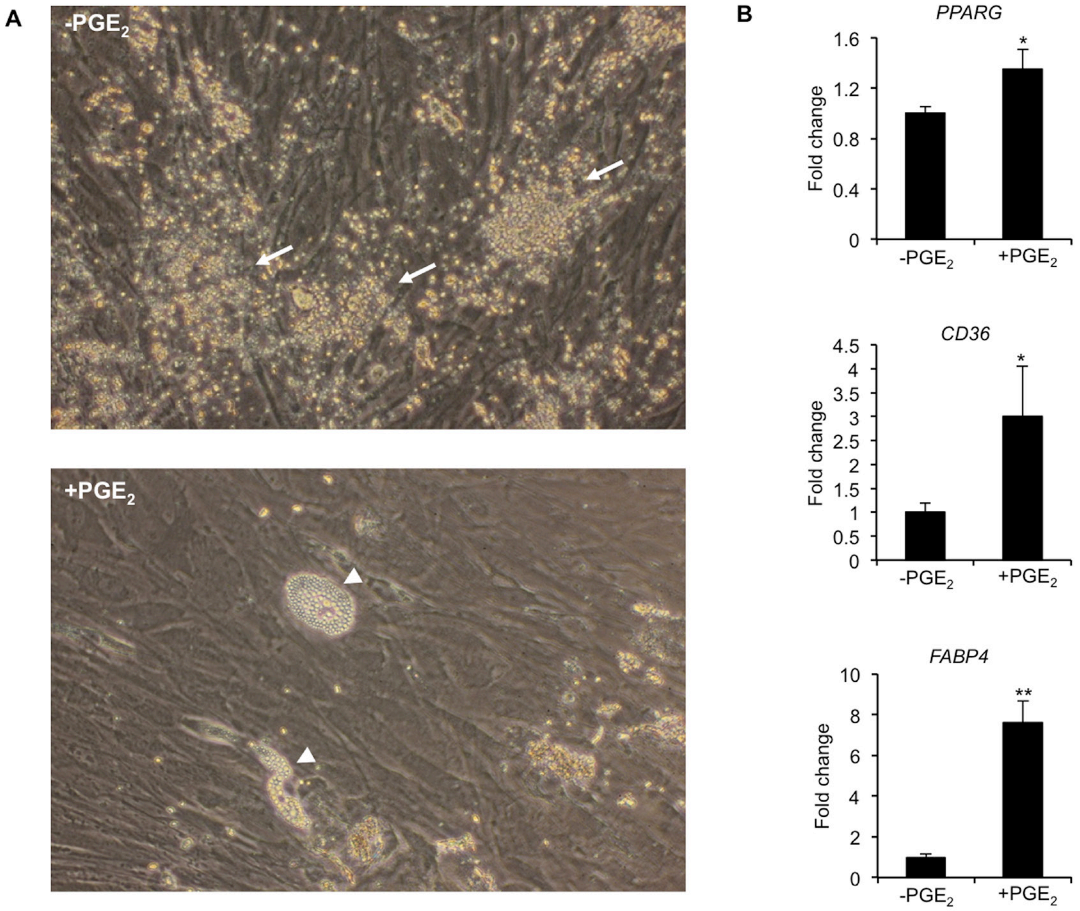

Supplementary Figure S3. PGE_2 stimulates adipogenic differentiation of hBMSCs maintained under osteogenic culture conditions. (**A**) Phase contrast images of hBMSCs continuously treated for 16 days in osteogenic medium in the absence (-PGE_2) or presence (+PGE_2) of PGE_2 (10 nM). Examples of mineralized deposits in untreated hBMSC cultures are identified with *white arrows*, and lipid-laden cells in PGE_2-treated hBMSC cultures are identified with *white arrow heads*. (**B**) RT-qPCR was used to determine expression levels of adipogenic markers *PPARG*, *CD36* and *FABP4* in hBMSCs at day 17 post-osteogenic induction. Data were normalized to *GUSB* and expressed as fold change as compared to non-induced controls at day 0 (value 1) using the comparative C_T method. $*p < 0.05$, $**p < 0.001$ as compared to untreated hBMSCs using ANOVA. The data represent triplicate determinations and were replicated at least two times. All values are presented as mean ± S.D.

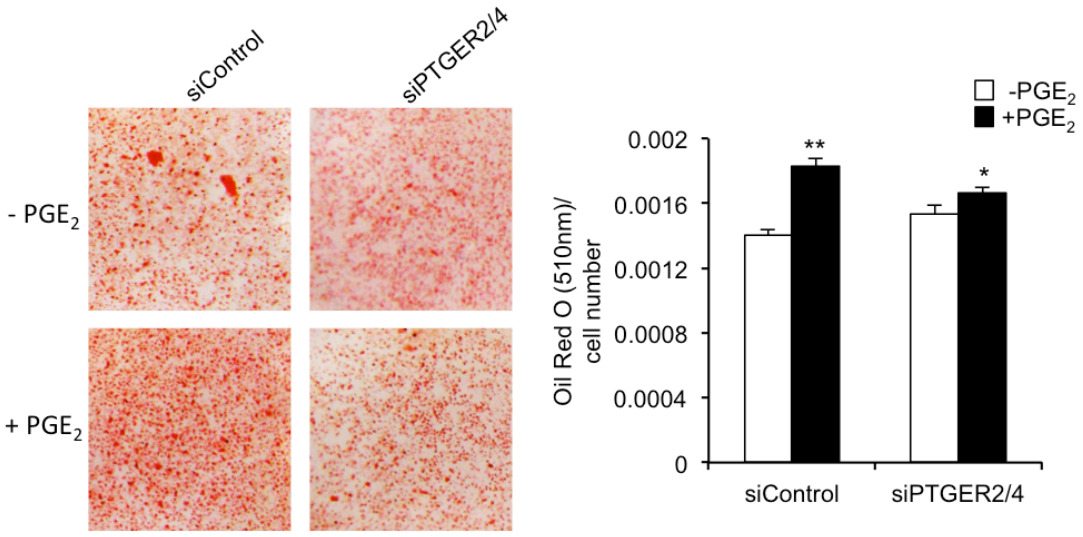

Supplementary Figure S4. PGE_2 effects on adipogenesis are mediated through PGE_2 receptor subtypes EP2 and EP4. The effects of continuous PGE_2 (10 nM) treatment on oil droplet accrual in siPTGER2 and siPTGER4 treated hBMSCs was assessed at day 15 by Alizarin Red S staining. *$p < 0.05$, **$p < 0.001$, as compared to untreated hBMSCs (-PGE_2) using Student's t-test. The data represent triplicate determinations of pooled samples from two biological replicates. All values are presented as mean ± S.D.

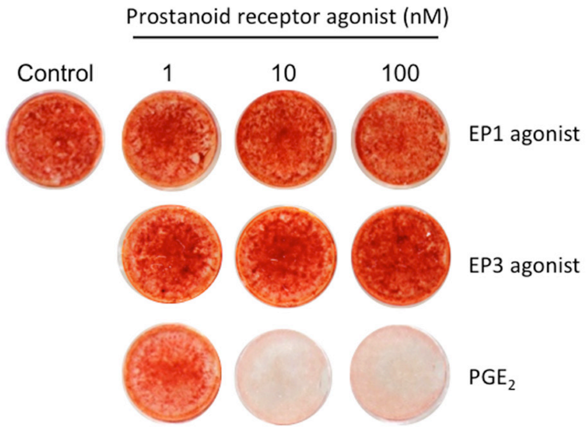

Supplementary Figure S5. Effect of PGE_2 receptor agonists on hBMSC-mediated matrix mineralization. Alizarin Red S staining was used to assess the influence of varying concentrations of 7-Phenyl-trinor-prostaglandin E2 (EP1 agonist), Sulprostone (EP3 agonist), and PGE_2 on matrix mineralization in hBMSC cultures at day 14 post-osteogenic induction. The experiment was performed in triplicate and replicated at least two times.

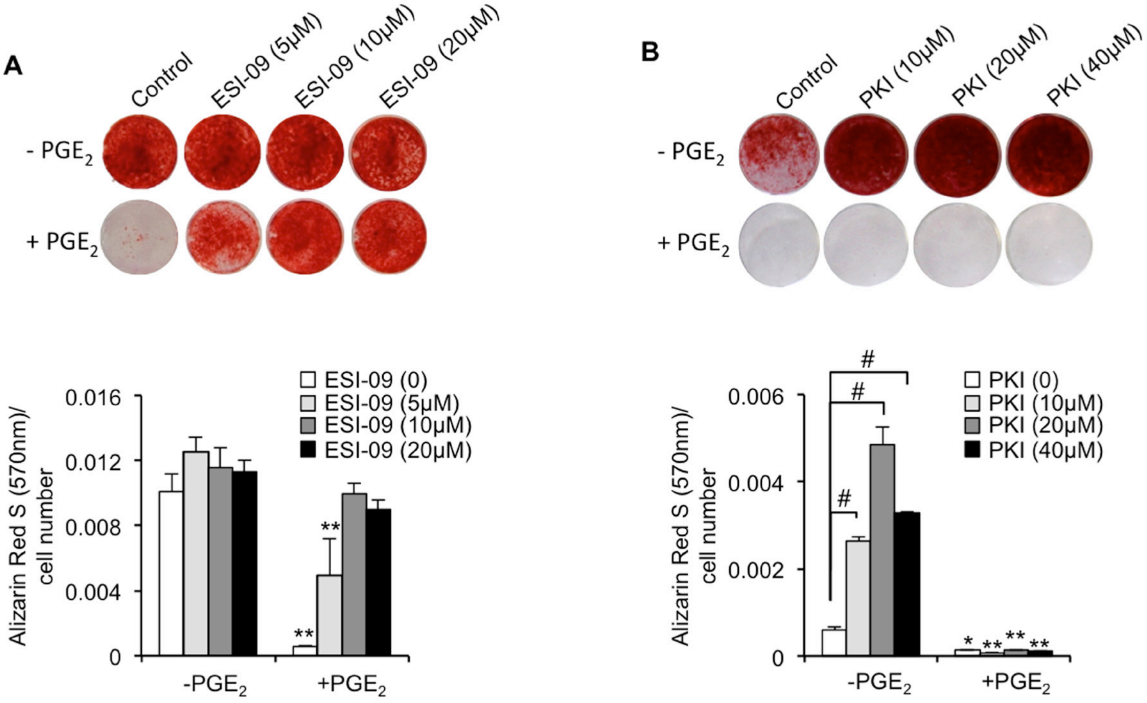

Supplementary Figure S6. Effects of ESI-09 and PKI on hBMSC-mediated matrix mineralization. hBMSCs were cultured continuously in the absence (-PGE2) or presence (+PGE_2) of PGE_2 (10 nM) with Epac inhibitor ESI-09 (**A**) or PKA inhibitor PKI (**B**) at varying concentrations, and matrix mineralization quantified at day 14 by Alizarin Red S staining. *$p < 0.05$, ** $p < 0.001$ as compared to untreated hBMSCs (–PGE_2); #$p < 0.001$ as compared to untreated hBMSCs (–PGE_2) in the absence of PKI using ANOVA. The data represent triplicate determinations and were replicated at least two times. All values are presented as mean ± S.D.

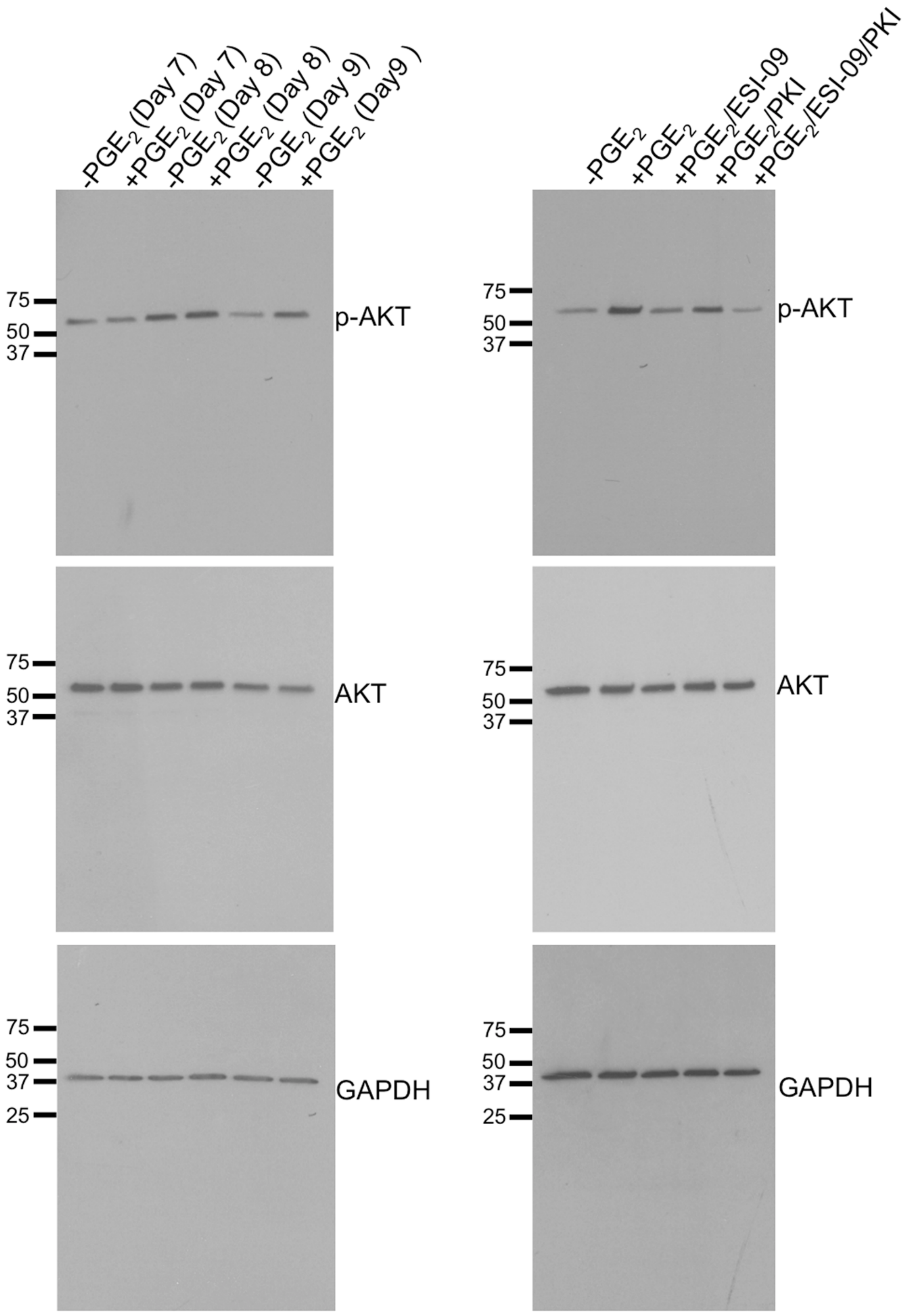

Supplementary Figure S7. Uncropped images of Western blot results used in Figure 7 of main manuscript.
